# Early Sensory Deprivation Leads to Differential Inhibitory Changes in the Striatum During Learning

**DOI:** 10.3389/fncir.2021.670858

**Published:** 2021-05-28

**Authors:** Nihaad Paraouty, Todd M. Mowery

**Affiliations:** ^1^Center for Neural Science, New York University, New York, NY, United States; ^2^Department of Otolaryngology, Head and Neck Surgery, Rutgers Robert Wood Johnson Medical School, New Brunswick, NJ, United States; ^3^Rutgers Brain Health Institute, Rutgers University, New Brunswick, NJ, United States

**Keywords:** corticostriatal pathway, associative learning, auditory discrimination, medium spiny neuron, layer 5 neurons, synaptic inhibition, hearing loss

## Abstract

The corticostriatal circuit has been identified as a vital pathway for associative learning. However, how learning is implemented when the sensory striatum is permanently impaired remains unclear. Using chemogenetic techniques to suppress layer five auditory cortex (AC) input to the auditory striatum, learning of a sound discrimination task was significantly impacted in freely moving Mongolian gerbils, in particular when this suppression occurs early on during learning. Whole-cell recordings sampled throughout learning revealed a transient reduction in postsynaptic (GABAA) inhibition in both striatal D1 and D2 cells in normal-hearing gerbils during task acquisition. In contrast, when the baseline striatal inhibitory strengths and firing rates were permanently reduced by a transient period of developmental sensory deprivation, learning was accompanied by augmented inhibition and increased firing rates. Direct manipulation of striatal inhibition *in vivo* and *in vitro* revealed a key role of the transient inhibitory changes in task acquisition. Together, these results reveal a flexible corticostriatal inhibitory synaptic plasticity mechanism that accompanies associative auditory learning.

## Introduction

The ability of an organism to associate different stimuli from the environment with specific sets of actions is fundamental to survival. Evidence from a range of species suggests that the corticostriatal network governs the acquisition of goal-directed behaviors (Balleine et al., 2007; Balleine and O’Doherty, 2010; Dolan and Dayan, 2013; Reig and Silberberg, 2014; Sippy et al., 2015; Yartsev et al., 2018; Cox and Witten, 2019), and reward-based learning in general (Wickens et al., 2003, 2007; Calabresi et al., 2007; Thorn et al., 2010; Humphries et al., 2012; Kupferschmidt et al., 2017). Degeneration in the corticostriatal network is linked to a spectrum of neurological and neuropsychiatric disorders, such as autism spectrum, Huntington’s, schizophrenia, Parkinson’s, amyotrophic lateral sclerosis, and obsessive-compulsive disorder (Shepherd, 2013), which are often accompanied by various impairments in action control and reward-related processes.

The posterior tail of the dorsal striatum, termed the auditory striatum plays a key role in sound-action associations (Znamenskiy and Zador, 2013; Xiong et al., 2015; Chen et al., 2019; Guo et al., 2019). In fact, the auditory striatum receives a majority of its excitatory inputs from the auditory cortex (AC; McGeorge and Faull, 1989; Voorn et al., 2004; Budinger et al., 2008; Hackett, 2011; Mowery et al., 2017). More precisely, the AC-auditory striatum circuit has been shown to be critical for sound discrimination (Znamenskiy and Zador, 2013), and optogenetic activation or silencing of auditory striatal neurons can bias discrimination performances (Guo et al., 2018). Like the rest of the basal ganglia, the auditory striatum includes two distinct populations of medium spiny neurons (MSNs), defined in part by the expression of dopamine receptor type: D1-receptor expressing (direct pathway) and D2-receptor expressing cells (indirect pathway). These MSNs receive, in majority excitatory input from AC layer 5 intratelencephalic neurons (IT) and pyramidal tract neurons (PT), respectively (Reiner et al., 2010; Cui et al., 2013; Freeze et al., 2013; Kress et al., 2013; Calabresi et al., 2014; Cazorla et al., 2014; Rock et al., 2016). In the classical model, the direct pathway is associated with reinforcing movement and locomotion, while the indirect pathway is linked to freezing and movement suppression (Cox and Witten, 2019). However, their respective roles in learning an auditory discrimination task remain unclear, especially when the auditory striatum is permanently impaired.

At a cellular level, learning is often associated with a transient downregulation in GABAergic inhibition that facilitates long–term excitatory plasticity (eLTP) in cortical processing (Wigström and Gustafsson, 1986; Steward et al., 1990; Mott and Lewis, 1991; Bilkey, 1996; Brucato et al., 1996; Cho et al., 2000; Ziakopoulos et al., 2000; Kreitzer and Malenka, 2008; Ormond and Woodin, 2011; Perugini et al., 2012). Similarly, auditory learning and eLTP in normal hearing models have been linked to transient decreases in inhibitory synaptic gain in layer 2/3 AC cells (Letzkus et al., 2011; Sarro et al., 2015), and in the perirhinal cortex (Kotak et al., 2017). However, the baseline inhibition in striatal cells is permanently disturbed with a transient period of developmental sensory deprivation (Mowery et al., 2017). Here, we asked whether auditory learning was accompanied by similar reductions in inhibition in such an impaired model of the corticostriatal pathway. Thus, using a combination of *in vivo* behavioral measures and *in vitro* recordings, we examined the changes in cellular and synaptic properties of layer 5 AC cells and their auditory recipient striatal D1 and D2 cells throughout learning of a Go-Nogo auditory discrimination task in control and developmental sensory-deprived Mongolian gerbils.

We first demonstrated the necessity of the corticostriatal pathway in learning a sound discrimination task by chemogenetically suppressing excitatory cortical input to auditory striatal D1 and D2 cells. As control animals transitioned from a naïve stage of poor discrimination performances to better discrimination performances, *in vitro* whole-cell recordings revealed a local and transient decrease in inhibitory post-synaptic strengths in D1 and D2 striatal cells. In contrast, we found that learning was accompanied by augmented inhibition in D1 and D2 striatal cells of developmental sensory-deprived animals. By direct manipulation of inhibitory levels during task acquisition, we found that learning could be suppressed in control animals when inhibition was maintained at a high level through local infusions of a GABAA-α2/3 subunit receptor agonist. Together, these results bridge the gap between control and pathological corticostriatal networks by showing that reduced inhibition might not be the only facilitating factor for auditory associative learning in the corticostriatal network. Our results suggest that transient changes to the inhibitory tone in striatal D1 and D2 cells may be required for learning-related plasticity to occur. Such transient and flexible inhibitory shifts in both striatal D1 and D2 cells may be key for reward-based auditory learning.

### Materials and Methods

#### Experimental Animals

Gerbil (*Meriones unguiculatus*) pups were weaned at postnatal day (P) 30 from commercial breeding pairs (Charles River). Littermates were caged together, but separated by sex, and maintained in a 12 h light/dark cycle. All procedures related to the maintenance and use of animals were approved by the University Animal Welfare Committee at New York University. Both male and female gerbils were tested (*n* = 109 gerbils, 67 female).

#### Reversible Auditory Deprivation

Mild auditory deprivation was induced by inserting a malleable plug (BlueStik Adhesive Putty, RPM International Inc.) into the opening of each ear canal at P11 (Mowery et al., 2014, 2017; Caras and Sanes, 2015). Animals were checked daily, and earplugs were adjusted to accommodate growth. Earplugs were removed at P35. Earplugs attenuate auditory brainstem responses and perceptual thresholds by approximately 15–50 dB, depending on frequency, and the attenuation is completely reversible (Mowery et al., 2014; Caras and Sanes, 2015).

#### Behavioral Setup

Gerbils were placed in a plastic test cage (dimensions: 0.25 × 0.25 × 0.4 m for 62 animals and 0.4 × 0.4 × 0.4 m for 42 animals) that was housed in a sound attenuation booth (Industrial Acoustics; internal dimensions: 2.2 × 2 × 2 m), and observed *via* a closed-circuit monitor. Auditory stimuli were delivered from a calibrated free-field tweeter (DX25TG0504; Vifa) positioned 1 m above the test cage. Sound calibration measurements were made with 1/4 inch free-field condenser recording microphone (Bruel and Kjaer). A pellet dispenser (Med Associates Inc., 20 mg) was connected to a food tray placed within the test cage, and a nose port was placed on the opposite side. Stimuli, food reward delivery, and behavioral data acquisition were controlled by a personal computer through custom MATLAB scripts and an RZ6 multifunction processor (Tucker-Davis Technologies).

#### Sound Stimuli

The Go stimulus consisted of amplitude modulated (AM) frozen broadband noise tokens (25 dB roll-off at 3.5 kHz and 20 kHz) with a modulation rate of 12 Hz and a modulation depth of 100%. The Nogo stimulus was similar to the Go stimulus, except for the modulation rate which was 4 Hz. Both Go and Nogo stimuli had a 200 ms onset ramp, followed by an unmodulated period of 200 ms which then transitioned to an AM stimuli. The sound level used was 66 dB SPL.

#### Behavioral Training

Animals were placed on controlled food access and trained using an appetitive reinforcement operant conditioning procedure. When introduced to the test cage, animals first learned to eat food pellets (Bio Serv) placed in the food tray. After this phase, the Go stimulus (12 Hz AM, 100% modulation depth) was delivered whenever animals were at the food tray. Animals were then trained to respond to the Go stimulus by approaching the food tray. After this sound-food association phase, the nose port was placed in the testing cage. During the first day of nose port training, the experimenter triggered trials whenever animals were in close proximity to the port. This maximized exploration of the nose port and facilitated poking behavior. Within 1 to 2 training sessions, animals were shaped to reliably initiate Go trials by placing their nose in the port, without any experimenter intervention. During the nose port training sessions, only Go stimuli were presented. Once animals reached a hit rate >80% and were performing a minimum of 80 Go trials, Nogo trials were introduced and the Go-Nogo phase began.

At this point, animals were run once per day until they performed at least 80 Go trials and at least 20 Nogo trials. Typically, a session lasted on average 30 min (min-max: 20–50 min). During Go trials, responses were scored as a Hit when animals approached the food tray and broke a light beam to obtain a food reward. If animals re-poked or did not respond during the 5-s time window following a Go stimulus, then it was scored a Miss. During Nogo trials, responses were scored as a False Alarm when animals incorrectly approached the food tray and broke the light beam. If animals re-poked or did not respond during the 5-s time window following a Nogo stimulus, then it was scored a Correct Reject. On the second day of Nogo training, False Alarm trials were paired with a 2-s time out, during which the house lights were extinguished and the animal could not initiate a new trial. From day 3 onwards, a 4-s time out was used when animals False Alarmed. The presentation of Go and Nogo trials was randomized to avoid animals developing a predictive strategy. Hit and False Alarm rates were constrained to floor (0.05) and ceiling (0.95) values. A performance metric, d prime (d′) was calculated for each session by performing a z-transform of both Hit rate and False Alarm rate: d′ = z(Hit rate) − z(False Alarm rate) (Green and Swets, 1966).

**Figure 1:**
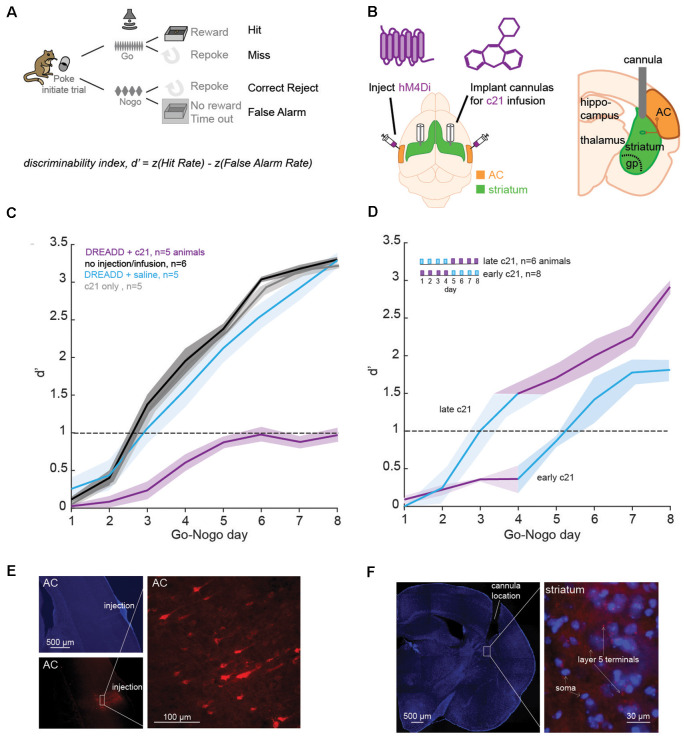
Suppression of the corticostriatal pathway impacts learning. **(A)** Go-Nogo discrimination task. A performance measure, d′ is computed for each animal and for each Go-Nogo session. **(B)** Illustration of the surgical procedure in which bilateral injections of iDREADD are performed in the auditory cortex (AC), followed by cannulae insertions in the auditory striatum. Local infusions of the activating agent, c21, or saline were carried out prior to behavioral testing. **(C)** Mean (±SEM) d′ measures across Go-Nogo days for different groups tested: (1) in purple, animals with iDREADD injections and c21 infusions, (2) in black, animals without iDREADD injections and without any infusion, (3) in blue, animals with iDREADD injections and saline infusions, and (4) in gray, animals without iDREADD injections but with c21 infusions. **(D)** Mean (±SEM) d′ measures for two additional groups: (1) late c21 animals with iDREADD injections and saline infusions on days 1–4, followed by c21 infusions on days 5–8, and (2) early c21 animals with iDREADD injections and c21 infusions on days 1–4, followed by saline infusions on days 5–8. **(E)** Photomicrographs confirming the injection site of iDREADD with labeled AC cells (mCherry). **(F)** Confirmation of the position of implanted cannulae in the dorsolateral striatum with an overlay of MSNs (in blue, DAPI) and AC projections (in red, mCherry).

Three different phases of learning were described, based on the results from Figure 1. First, a *naïve phase* was described as d′ criteria values <1 as the iDREADD + c21 animals (Figure 1C, purple line) showed a d′ below 1 across the eight tested days. Next, an *acquisition phase* was described based on the results from Figure 1D (late c21 group). Once those animals were performing with a d′ > 1, c21 infusions did not decrease their performance below 1. Thus the acquisition phase was defined here for d′ values comprised between 1 and 2. Last, a *mastery phase* was defined as the range of values closest to the highest d′ value, which is limited by the Hit and False Alarm rates. The latter was constrained to the floor (0.05) and ceiling (0.95) values. In order to account for variance between sessions, the mastery phase was defined for all d′ values >2.

#### Designer Receptors Exclusively Activated by Designer Drug Transfection

Gerbils were anesthetized (isoflurane 2%) and placed in a stereotaxic frame. The left and right temporal bone was exposed. A craniotomy was made in the temporal bone at the level of core AC (~3.9–3.2 mm rostral from lambda), and a durotomy was made around 3.5 and 3.3 mm rostral from lambda. A glass pipette was loaded with adenovirus containing a CaMKII promotor that transfects pyramidal neurons with the inhibitory DREADDs receptor HM4D (pAAV-CaMKIIa-hM4D(Gi)-mCherry, Plasmid #50477). A Nanoject (Drummond) was then used to deliver 350 nL of the virus at a depth of 800–900 microns from the pial surface. Injections were made bilaterally. Histological feedback from our animals confirmed that the injection sites were consistently in Layer 5, although spread to other laminar layers did occur. Note that DREADDs activation occurred downstream through cannula perfusion of the activating drug. Finally, the craniotomy was covered with sterile bone wax, and the surgical site was closed with sutures.

#### Cannula Implantation

Gerbils were anesthetized (isoflurane 2%), placed in a stereotaxic frame, and the parietal, occipital, and frontal bones were exposed. The skin overlying these bones was removed and sinew were removed from the surface of the skull. Two anchor screws were placed over the frontal cortex and secured in place with dental acrylic (Hereaus). Two craniotomies were made for bilateral cannula insertion into striatal areas designated to receive dense input from AC layer 5 (~4.7 mm lateral and 3.7 mm rostral of lambda, see Mowery et al., 2017). Cannulae (Plastics One) were lowered to a depth of 3 mm from the skull surface and secured in place with dental acrylic (Hereaus). Dummy guide cannulae were inserted and protective caps were locked in place. Animals were allowed to recover for 1 week.

#### Cannula Infusions

Prior to all infusions, animals were anesthetized (~2% isoflurane). The concentration of the inhibitory-DREADD activating drug: Compound 21 (c21, HelloBio) was 5 mg/ml. Physiological saline (0.9% NaCl) was infused as indicated. The concentration of GABA_A_-α2/3 subunit agonist: TPA023 (Sigma) was 5 mg/ml. The dose of drugs and saline infused was 2 μl at a rate of 1 μl per minute. The dose remained unchanged for all animals across testing days. Following infusions, animals were allowed to fully recover in a recovery cage (for 15 min on average) before behavioral testing began. Higher doses of c21 produced noted behavioral, motor effects (like thigmotaxis and lethargy) suggesting both the effectiveness of the drugs and volumetric spread thresholds into the sensorimotor areas of the striatum.

#### Corticostriatal Brain Slice Preparation

Brain slices were obtained within 3 h after a training/testing session. The details for corticostriatal brain slice preparation have been previously described (see Mowery et al., 2017). Animals were deeply anesthetized (chloral hydrate, 400 mg/kg, IP) and brains dissected into 4°C oxygenated artificial cerebrospinal fluid (ACSF, in mM: 125 NaCl, 4 KCl, 1.2 KH_2_PO_4_, 1.3 MgSO_4_, 26 NaHCO_3_, 15 glucose, 2.4 CaCl_2_, and 0.4 L-ascorbic acid; and bubbled with 95%O_2_-5%CO_2_ to a pH = 7.4). A 25° cut was made through the right hemisphere and the brains were vibratome-sectioned through the left hemisphere to obtain 300–400 μm perihorizontal auditory corticostriatal slices. To validate the thalamo-recipient AC, a bipolar stimulating electrode (FHC) was placed at the rostral border of the medial geniculate (MG), and MG-evoked field responses were recorded in the AC. To validate cortico-recipient striatum, a bipolar stimulating electrode was placed in layer 5 AC and AC-evoked field responses were recorded in the striatum. Whole-cell current clamp recordings were obtained (Warner PC-501A) from striatal MSNs at 32°C in oxygenated ACSF. Recording electrodes were fabricated from borosilicate glass (1.5 mm OD; Sutter P-97). The internal recording solution contained (in mM): 5 KCl, 127.5 K-gluconate, 10 HEPES, 2 MgCl_2_, 0.6 EGTA, 2 ATP, 0.3 GTP, and 5 phosphocreatine (pH 7.2 with KOH). The resistance of patch electrodes filled with an internal solution was between 5 and 10 MΩ. Access resistance was 15–30 MΩ, and was compensated by about 70%.

Recordings were digitized at 10 kHz and analyzed offline using custom Igor-based macros (IGOR, WaveMetrics, Lake Oswego, OR, USA). All recorded neurons had a resting potential ≤−50 mV and overshooting action potentials. Frequency-current (F-I) curves were constructed from the responses to 1,500 ms current pulses, in steps of 100 pA (Mowery et al., 2014). Inhibitory postsynaptic potentials (IPSP) were evoked *via* biphasic stimulation of local fast-spiking interneurons for striatal neurons (1–10 mV, 10 s interstimulus interval) in the presence of ionotropic glutamate receptor antagonists (6,7-Dinitroquinoxaline-2,3-dione, DNQX, 20 μM; 2-amino-5-phosphonopentanoate, AP-5, 50 μM). The drugs were applied for a minimum of 8 min before recording IPSPs. Importantly, all recordings were systematically carried out at 200–300 microns from the right shank of the biphasic stimulator. In addition, the depth of recordings were carried out in the first 15–25 microns of tissue as visibility quickly decreases in striatal tissue under IRDIC illumination. To control for differences in stimulation strengths, we systematically employed 0.3–0.4 mA of stimulation to obtain a plateau in IPSP amplitudes. Once this maximum was reached, increasing stimulation did not lead to further increases in amplitude or duration for both GABA-A/B potentials. In addition, pilot studies demonstrated that higher stimulation levels tend to damage the surrounding tissue and lead to local circuit changes (results not shown here). Peak amplitudes of the short latency hyperpolarization (putative GABAA component) were measured from each response at a holding potential (V_hold_) of −50 mV. In a subset of experiments (*n* = 10), we verified that the short-latency IPSP components were selectively blocked by a GABAA antagonist (20 μM bicuculline), thereby suggesting that the IPSPs reported in this study are related to GABAA receptor potentials.

#### Assessing the Suppression Effect of hM4Di-DREADD Activating Drug

Proof of principle experiments for inhibitory DREADD inhibitory action were conducted in animals (*n* = 2) that had received unilateral injections of iDREADD 2–3 weeks prior to corticostriatal slice preparation (see Supplementary Figure 2). For these experiments, whole cell recordings were made from medium spiny cells (current clamp: −80 mV hold). Excitatory postsynaptic potentials were evoked by stimulating layer 5 AC pyramidal cells with a biphasic stimulating electrode. Cells were held at −80 mV to isolate AMPA receptor potentials. After establishing a baseline, cells were exposed to 30 min of ACSF containing the iDREADD activating drug: Compound 21 (50 uM, HelloBio). Excitatory post synaptic potentials (EPSPs) were collected up to an hour after drug exposure prior to washout.

#### Histology

At the end of experiments all implanted animals were deeply anesthetized with an intraperitoneal injection of sodium pentobarbital (150 mg/kg) and perfused with phosphate-buffered saline and 4% paraformaldehyde. Brains were extracted, post-fixed, and sectioned at 50 μm on a benchtop vibratome (Leica). Sections were stained for DAPI (4′,6-diamidino-2-phenylindole), and coverslipped for imaging. DAPI images were acquired at 2×, 10× and 40× using a revolve microscope (Echo) and locations of cannulae were verified and compared to a gerbil brain atlas (Radtke-Schuller et al., 2016). For the animals which received both iDREADD injections and bilateral cannulae implants, both brightfield and fluorescent images were acquired to confirm virus expression in AC and projections to the auditory striatum.

#### Statistical Analyses

Statistical tests for distribution and significance were performed using the SAS-based package JMP. Normality was determined using the Shapiro–Wilk Test. Groups with normally distributed data were analyzed using a mixed-model ANOVA, as indicated. Tukey’s HSD comparisons were used as indicated for pairwise comparisons. Nonparametric statistical tests were used when data was not normally distributed (Wilcoxon tests).

#### Results

##### Necessity of the Corticostriatal Pathway in Learning a Sound Discrimination Task

We first assessed the necessity of the corticostriatal pathway in learning a Go-Nogo sound discrimination task in freely-moving Mongolian gerbils (Figure 1A). Specifically, we chemogenetically suppressed the excitatory input from layer 5 AC to the auditory striatum with an inhibitory Designer Receptors Exclusively Activated by Designer Drug (iDREADD; Supplementary Figure 1). To suppress both D1 and D2 pathways, we bilaterally injected hM4Di-mCherry, an inhibitory DREADD into AC layer 5 to express hM4Di receptor in IT and PT neurons (Figure 1B). The hM4Di receptor hyperpolarizes the cell (i.e., increases potassium influx), and decreases the presynaptic excitability, thereby reducing the probability of presynaptic glutamatergic release (see Supplementary Figure 2). To limit iDREADD activation to different projecting sites of IT and PT cells, we implanted bilateral cannulae in the auditory striatum for local infusions of the activating agent, compound 21 (c21, Figure 1B). After a week of recovery, animals began the behavioral task. Briefly, animals were trained to initiate each trial by entering a nose-port which triggered the presentation of the Go stimulus: a 12-Hz amplitude-modulated noise (AM), signaling the availability of a food pellet. Once animals were performing >80 Go trials, with a hit rate above 80%, we proceeded to the Go-Nogo phase of the task (Figure 1A). The Go stimulus (12-Hz AM noise) remained unchanged and indicated the presence of a food reward, while the Nogo stimulus (4-Hz AM noise) signaled the absence of a food reward. A discrimination performance metric, d-prime (d′) was calculated for each session as d′ = z(hit rate) − z(false alarm rate).

In order to suppress the corticostriatal circuit, animals received bilateral injections of iDREADD in AC layer 5 and infusions of c21 in the auditory striatum on all Go-Nogo days (*n* = 5; Figure 1C, purple line). Three control conditions were run, the first one consisted of animals without iDREADD injections nor c21 infusions (*n* = 6; black line). The second control group was composed of animals which received bilateral injections of iDREADD and infusions of saline on all Go-Nogo days (*n* = 5; blue line). Finally, the third control group was composed of animals which only received infusions of c21 (*n* = 5; gray line). A significant group effect was found (mixed model ANOVA, *F*_(3,16)_ = 36.76, *p* < 0.001), with the iDREADD + c21 group (purple line) showing significantly poorer task acquisition as compared to the three control groups (all Bonferroni corrected *post hoc* comparisons, *p* < 0.001). In contrast, the three control groups were not significantly different from one another (*p* > 0.05 for all *post hoc* comparisons).

To further identify the necessity of the corticostriatal pathway at different stages of the learning process, we ran two additional groups of animals. In the first condition, animals received bilateral injections of iDREADD and infusions of c21 on the first 4 days of Go-Nogo, followed by saline infusion on the last 4 days (*early c21*, *n* = 8; Figure 1D). In parallel, animals in the second condition received bilateral injections of iDREADD and infusions of saline on the first 4 days of Go-Nogo, followed by c21 infusion on the last 4 days (*late c21*, *n* = 4; Figure 1D). Early c21 infusions caused a significant learning delay as compared to the three control groups from Figure 1C (mixed model ANOVA, all Bonferroni corrected *post hoc* group comparisons, *p* < 0.001). In contrast, late c21 infusions resulted in no significant group difference as compared to the three control groups from Figure 1C (*p* > 0.05 for all *post hoc* comparisons). Once c21 infusions were replaced by saline infusions in the early c21 group, the mean d′ measure increased above 1.0, showing that c21 infusions early on did not permanently inhibit learning.

Comparison of all groups tested showed no significant difference in terms of latency of response (one-way ANOVA, Kruskal–Wallis *H* test: $\chi_{\left(5\right)}^{2}$ = 1.45, *p* = 0.919) nor in terms of the total number of trials performed during each session (one-way ANOVA, Kruskal–Wallis *H* test: $\chi_{\left(5\right)}^{2}$ = 2.97, *p* = 0.704), suggesting that the learning differences reported above could not be explained by a motor deficit. Transfection and cannulae positions were confirmed for each animal at the end of each experiment (Figures 1E,F). Together, these results suggest that suppressing the corticostriatal pathway, in particular early on during learning prevented the acquisition of an auditory discrimination task (i.e., behavioral d′ remained below 1).

##### Learning Is Accompanied by a Transient Change in Inhibition in Control Animals

**Figure 2:**
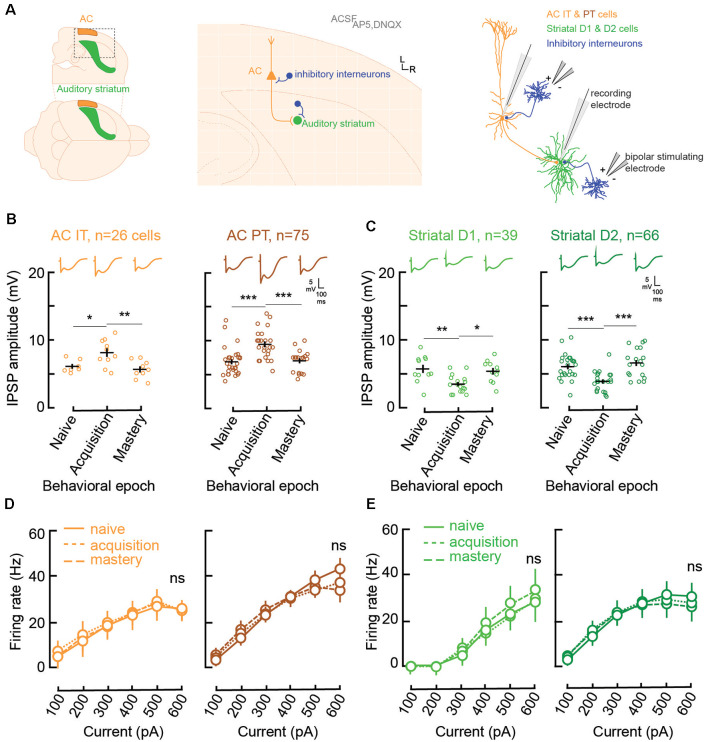
Learning is accompanied by changes in both AC and striatal GABAA inhibitory strengths. **(A)** Illustration of slice preparation for recordings in AC layer 5 IT and PT cells, and striatal D1 and D2 MSNs. Whole-cell current was used to measure inhibitory postsynaptic strength (IPSP) amplitudes and evoked firing rates (see “Materials and Methods” section). **(B)** Individual (open circles) and mean (±SEM, black lines) maximum evoked IPSP amplitudes in AC layer 5 IT and PT cells from normal-hearing gerbils (*n* = 23) previously trained until their performance matched one of three behavioral epochs: naïve (d′ < 1), acquisition (d′ between 1 and 2), and mastery phase (d′ > 2). Representative traces are shown at the top. **(C)** Individual and mean (±SEM) IPSP amplitudes in striatal D1 and D2 cells as a function of behavioral epochs. **(D)** Input-output functions (firing rate patterns; mean ± SEM) for IT and PT cells as a function of behavioral epochs. **(E)** Input-output functions for striatal D1 and D2 cells as a function of behavioral epochs. Asterisks denote statistically significant differences at the following levels: *p < 0.05, **p < 0.01, ***p < 0.001, and ns = not significant.

As the corticostriatal pathway is crucial for auditory associative learning, it is likely that such learning is supported by changes in cellular and synaptic properties of layer 5 AC cells and striatal D1 and D2 MSNs in the control (i.e., normal-hearing) model. More precisely, excitatory long-term potentiation (eLTP) associated with auditory learning has been shown to be facilitated by GABAergic inhibition in many brain regions (Cho et al., 2000; Letzkus et al., 2011; Perugini et al., 2012; Sarro et al., 2015; Kotak et al., 2017). Here, we applied a cross-sectional approach to investigate how synaptic inhibitory strengths (GABAA receptor potentials, see “Materials and Methods” section) along the direct and indirect pathways change as a function of learning. Following each day of behavioral Go-Nogo testing, an animal (*n* = 23) was randomly selected to undergo corticostriatal functional slice preparation, followed by whole cell current-clamp recordings of both AC layer 5 IT and PT cells, as well as their respective MSN targets, D1 and D2 cells (Figure 2). As described in Mowery et al. (2017), the cells were clustered using their electrophysiological properties (Kawaguchi, 1993; Cepeda et al., 2008; Gertler et al., 2008; Mowery et al., 2017; Goodliffe et al., 2018; see Supplementary Figure 3). The AC cell phenotype was characterized by cell type-specific discharge properties (Hattox and Nelson, 2007; Mowery et al., 2017; see Supplementary Figure 3). The results are presented in Figure 2 for both AC IT and PT cells and auditory striatal D1 and D2 cells during three phases of learning characterized by different d′ criteria values based on the results from Figures 1C,D (see “Materials and Methods” section): a naïve phase: d′ < 1, an acquisition phase: d′ between 1 and 2, and mastery phase: d′ > 2.

Both IT (*n* = 26) and PT (*n* = 75) cells showed significant increases in their inhibitory postsynaptic strengths (IPSP amplitude, Figure 2B) when comparing the naïve phase with the acquisition phase, and a return to baseline during the mastery phase (IT naïve vs. acquisition, Tukey’s HSD comparisons, *p* = 0.034; IT acquisition vs. mastery, *p* < 0.005; PT naïve vs. acquisition, *p* < 0.001; PT acquisition vs. mastery, *p* = 0.0003). In contrast, for the striatal D1 (*n* = 39) and D2 cells (*n* = 66; Figure 2C), there was a significant decrease of IPSP amplitudes from the naïve phase to the acquisition phase, and a return to baseline during the mastery phase (D1 naïve vs. acquisition, *p* = 0.0023; D1 acquisition vs. mastery, *p* = 0.0108; D2 naïve vs. acquisition, *p* = 0.0003; D2 acquisition vs. mastery, *p* < 0.0001).

Unlike IPSP strength, there were no changes to the input-output functions, i.e., evoked firing rate patterns, during task acquisition (Figures 2D,E). For layer 5 AC, both IT and PT cells retained similar patterns of evoked firing rate throughout learning (IT naïve vs. acquisition, *p* = 0.61; IT acquisition vs. mastery, *p* = 0.67; PT naïve vs. acquisition, *p* = 0.51; PT acquisition vs. mastery, *p* = 0.81). Similarly, no change was found for firing rate patterns in D1 and D2 cells (D1 naïve vs. acquisition, *p* = 0.82; D1 acquisition vs. mastery, *p* = 0.264; D2 naïve vs. acquisition, *p* = 0.94; D2 acquisition vs. mastery, *p* = 0.61). Overall, normal auditory discrimination learning was not accompanied by changes in firing rate patterns of AC layer 5 cells nor auditory striatal cells. Conversely, auditory discrimination learning was accompanied by significant synaptic inhibitory changes, with a transient increase in IPSP strength in layer 5 AC and a transient decrease in IPSP strengths in both striatal D1 and D2 cells.

##### Augmented Inhibition Accompanies Learning in an Impaired Corticostriatal Model

Striatal function is permanently impacted by a transient period of sensory deprivation during development (Mowery et al., 2017). Precisely, when we compared cellular and synaptic properties of MSNs in a group of adult gerbils that received bilateral earplugs (to induce a conductive hearing loss) early during development, permanent physiological changes were found in terms of baseline firing rates and IPSP strengths, as compared to a control population (Mowery et al., 2014, 2017; Caras and Sanes, 2015). Given those permanent shifts in inhibition, we tested whether such an impaired corticostriatal circuit was accompanied by similar changes in inhibition during learning as the control animals.

**Figure 3:**
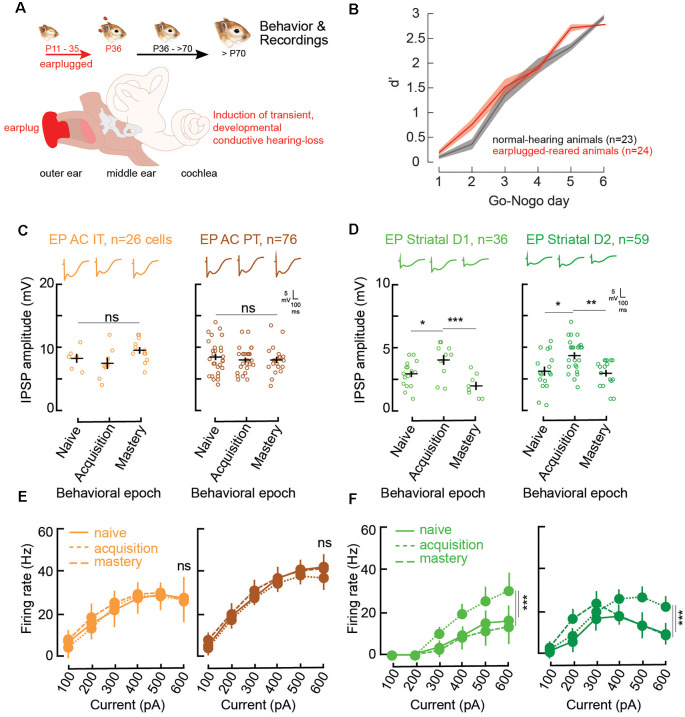
Impaired corticostriatal circuit supports learning through increases in striatal inhibitory strengths and firing rates. **(A)** Illustration of the procedure for the induction of developmental auditory deprivation in gerbils. Animals were earplugged (EP) from P11 to P35. Behavioral testing and recordings began only from P70. **(B)** Mean (±SEM) d′ measures across Go-Nogo days for the control population (gray, *n* = 23) and the EP animals (red, *n* = 24). **(C)** Individual (open circles) and mean (±SEM, black lines) maximum evoked IPSP amplitudes of AC layer 5 IT and PT cells as a function of behavioral epochs for the EP animals. Representative traces are shown at the top. **(D)** Individual and mean (±SEM) IPSP amplitudes in striatal D1 and D2 cells as a function of behavioral epochs for the EP animals. **(E)** Input-output functions (firing rate patterns; mean ± SEM) for IT and PT cells as a function of behavioral epochs for the EP animals. **(F)** Input-output functions for striatal D1 and D2 cells as a function of behavioral epochs for the EP animals. Asterisks denote statistically significant differences at the following levels: *p < 0.05, **p < 0.01, ***p < 0.001, and ns = not significant.

To achieve this, a group of gerbils received bilateral earplugs from the day of ear canal opening (postnatal day, P11) until the beginning of the juvenile phase of development (P35). As from P36, the earplugged reared animals (EP, *n* = 24) were allowed to recover under normal-hearing conditions (Figure 3A). The EP animals were trained in a similar manner as the control animals to perform the Go-Nogo sound discrimination task. The individual and mean performance of both groups of animals are shown in Figure 3B (control animals in gray and EP animals in red). The performance of both groups of animals was not statistically different (mixed model ANOVA, group effect: *F*_(1,25)_ = 0.253, *p* = 0.62). No significant difference was found in terms of age (one-way ANOVA, Kruskal–Wallis *H* test: $\chi_{\left(1\right)}^{2}$ = 3.16, *p* = 0.075), or the number of trials performed per day in each group ($\chi_{\left(1\right)}^{2}$ = 1.32, *p* = 0.250). Similarly, no significant group difference was found in terms of response latency ($\chi_{\left(1\right)}^{2}$ = 0.05, *p* = 0.823).

As learning was not impacted by a transient developmental hearing loss, we asked whether similar inhibitory synaptic changes in AC and auditory striatal cells accompanied learning in the EP animals as the control ones. Hence, whole cell recordings of AC layer 5 IT (*n* = 26) and PT cells (*n* = 76), as well as their projection target D1 (*n* = 36) and D2 cells (*n* = 59) were also carried out for the EP animals at the three different phases of learning: naïve, acquisition, and mastery.

In line with Mowery et al. (2017), significant changes induced by the transient developmental auditory deprivation were present in *adult* striatal D1 and D2 cells. More precisely, there were significant reductions in baseline inhibitory strength for striatal D1, and D2 cells (Figures 2C, 3D, naïve stage; baseline control vs. baseline EP, *p* = 0.0006 and *p* < 0.0001, respectively). In addition, significant changes in baseline evoked firing rate patterns in D1 and D2 cells were present more than 30 days after hearing was restored (Figures 2E, 3F, naïve stage; baseline control vs. baseline EP, *p* = 0.0231 and *p* < 0.0001, respectively).

During the course of learning, in contrast to the control population, no significant changes in IPSP amplitudes were found for AC IT and PT cells in the EP group (Figure 3C; IT naïve vs. acquisition, Tukey’s HSD corrected *post hoc* comparisons, *p* = 0.760; IT acquisition vs. mastery, *p* = 0.093; PT naïve vs. acquisition, *p* = 0.736; PT acquisition vs. mastery, *p* = 1.000). While transient decreases in IPSP amplitudes were found for control striatal cells during learning, in the EP group, significant increases in inhibitory strength were observed for both D1 and D2 cells during task acquisition (Figure 3D; D1 naïve vs. acquisition, *p* = 0.029; D2 naïve vs. acquisition, *p* = 0.0106). Once the EP animals mastered the task, the IPSP amplitudes returned to baseline (D1 acquisition vs. mastery, *p* = 0.0007; D2 acquisition vs. mastery, *p* = 0.0034).

Moreover, there were no significant firing rate changes in AC with learning in the EP animals (Figure 3E; IT naïve vs. acquisition, *p* = 0.820; IT acquisition vs. mastery, *p* = 0.468; PT naïve vs. acquisition, *p* = 0.232; PT acquisition vs. mastery, *p* = 0.130). Conversely, significant changes in the evoked firing rate patterns of EP striatal D1 and D2 cells were found with learning (Figure 3F). More specifically, a significant increase in firing rate was observed during the acquisition phase for both D1 and D2 cells, and a return to baseline once the EP animals mastered the task (Figure 3F; D1 naïve vs. acquisition, *p* = 0.0018; D1 acquisition vs. mastery, *p* = 0.0032; D2 naïve vs. acquisition, *p* = 0.0003; D2 acquisition vs. mastery, *p* = 0.0015).

**Figure 4:**
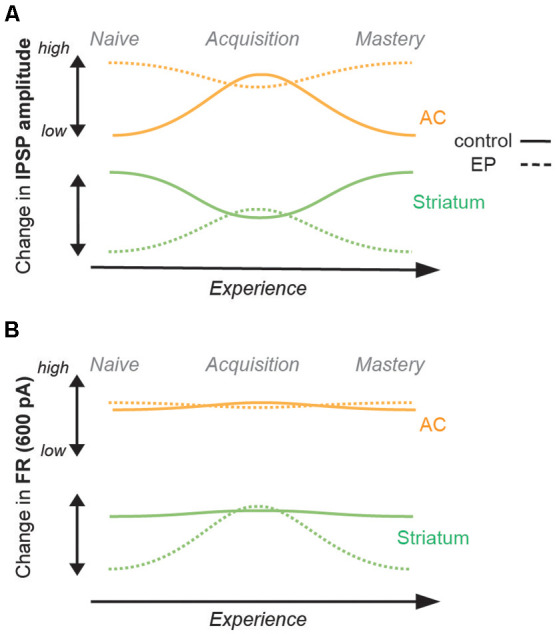
Summary of changes in inhibition and firing rates with learning. **(A)** Changes in IPSP amplitudes with learning for the control population (straight line), and for the EP population (dashed line). AC (IT and PT) data are shown in orange, and striatal (D1 and D2) data are shown in green. While a significant increase was found in IPSP amplitude of AC cells (both IT and PT cells) in the control population, no significant change was found for the EP group. A significant decrease was found in the IPSP amplitude of striatal cells (both D1 and D2 cells) in the control population, but in contrast, a significant increase was found for the EP group. **(B)** Changes in FR (Hz, 600 pA) with learning. No change in FR of AC cells was found in the control population, or in the EP group. No change in FR of striatal cells was found for the control population, but in contrast, a significant increase was found for the EP group.

Overall, in comparison to control animals, no changes in inhibitory strengths of layer 5 AC cells were found in EP animals (Figure 4A). However, significant changes were found in D1 and D2 cells, both in terms of IPSP amplitudes and firing rate patterns (Figures 4A,B). Furthermore, those transient changes both in IPSP amplitudes and firing rate patterns for D1 and D2 cells during learning move towards values close to the control population at the same stage (Supplementary Figures 4F–H).

Those results can be further explained by phenotype-dependent changes in the underlying cellular physiology of the EP animals. For D1 cells, a significant increase in adaptation ratio was found during task acquisition (Supplementary Figure 5D, D1 naïve vs. acquisition for EP, *p* = 0.0026; D1 acquisition vs. mastery, *p* = 0.0027). On the other hand, for D2 cells, significant changes in both resting membrane potential (more depolarized) and membrane resistance (higher) were observed (Supplementary Figure 5E, D2 naïve vs. acquisition for EP, *p* = 0.0042 and *p* < 0.0001; D2 acquisition vs. mastery, *p* = 0.0095 and *p* = 0.0019, respectively). In contrast, the underlying cellular physiology in control striatal D1 and D2 cells did not show any significant changes in resting membrane potential, membrane resistance, nor in sensory adaptation ratios throughout learning (Supplementary Figures 5A–C). Those transient shifts in the striatal cellular physiology of EP animals temporarily matched the cellular physiology of control animals during the task acquisition phase (comparison of control vs. EP for adaptation ratio in D1 cells, *p* = 0.5311; for resting membrane potential in D2 cells, *p* = 0.08; for membrane resistance in D2 cells, *p* = 0.538). Thus, through transient changes in adaptation ratios for D1 cells, and resting membrane potential and membrane resistance for D2 cells, the EP striatal MSNs seem to compensate for their phenotype-specific deficits and approach control values during learning. Together, these results suggest how plasticity could potentially be supported by the corticostriatal pathway when the baseline physiology is impaired.

##### Learning Is Causally Linked to Changes in Striatal Inhibition

**Figure 5:**
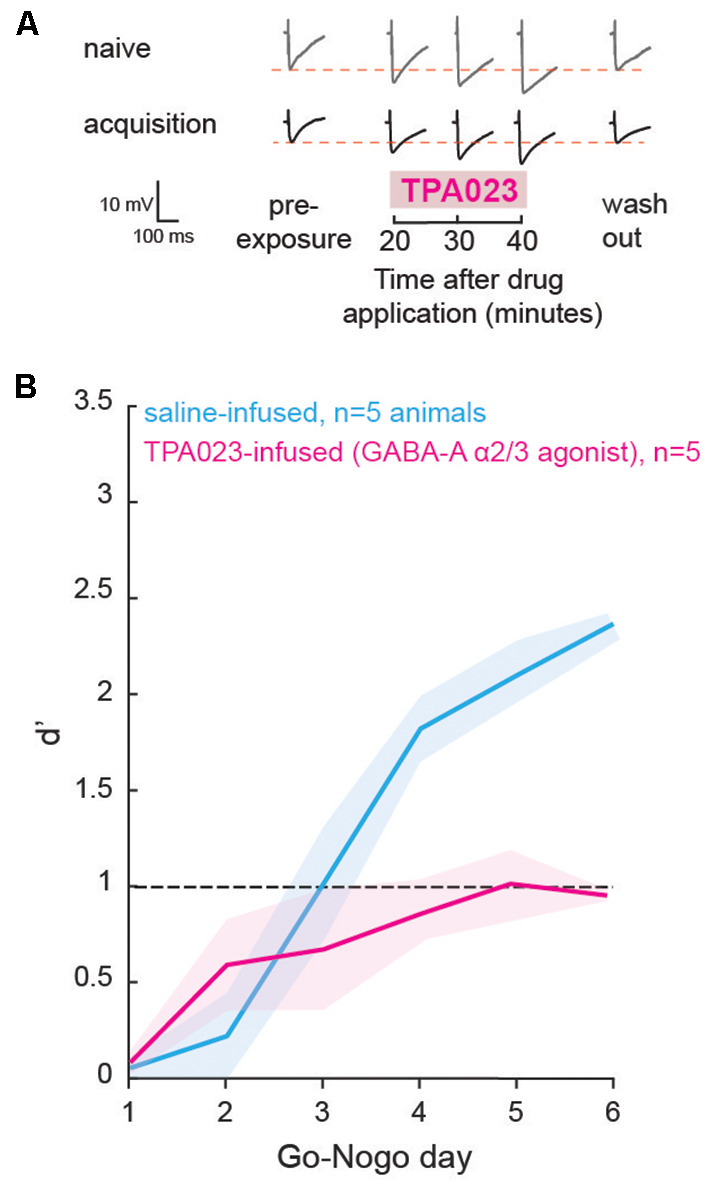
Direct manipulation of inhibition affected learning. **(A)** Representative traces of maximum evoked IPSPs recorded from striatal cells of the control population during the naïve and acquisition phases, with the addition of a GABAA-α2/3 subunit receptor agonist (TPA023) to the bath. Pre- and post-exposure traces are shown for comparison. **(B)** Mean (±SEM) d′ measures of animals which received local infusions of TPA023 (pink) or saline (blue) bilaterally in the auditory striatum prior to each Go-Nogo session. Infusions of TPA023 caused a significant impact on learning as compared to saline infusions.

In order to test whether the change in inhibition is causally related to behavioral task acquisition and learning, we used local infusions of selective GABAA agonists in the auditory striatum *in vivo*, prior to each Go-Nogo session. As we found a transient decrease in striatal inhibition during learning in the control population (Figure 2C), we predicted that maintaining a high level of inhibition would impact the rate of task acquisition. With a series of additional *in vitro* experiments, we first tested the sensitivity of striatal cells to a GABAA-α2/3 subunit receptor agonist: TPA023 (50 μM), as GABAA-α1 containing receptors, are not as widely expressed in the striatum (Hörtnagl et al., 2013). As expected, during both the naïve and acquisition phases, significant increases in IPSP amplitudes were found after application of TPA023 to the bath (Figure 5A; *p* = 0.001 and *p* = 0.004, respectively). Those results suggest constant sensitivity to TPA023 during learning. Thus, we predicted that daily infusions of TPA023 would lead to a significant delay in learning.

Prior to behavioral training, we implanted bilateral cannulae in the auditory recipient regions of the dorsolateral striatum for a subset of animals (*n* = 10). The animals were allowed to recover for a week and were then trained to perform a minimum of 80 Go trials correctly. Prior to each day of behavioral testing, a subset of animals (*n* = 5) received bilateral infusions of TPA023 (2 μl, 50 μM), and a second subset of animals (*n* = 5) received bilateral infusions of saline (0.9% NaCl, 2 μl). The performance d′ of both groups is shown in Figure 5B (TPA023-infused group in pink, and saline-infused group in blue). A mixed model ANOVA revealed significant group differences (ANOVA, *F*_(1,59)_ = 12.25, *p* = 0.0009), with the TPA023-infused animals being significantly delayed as compared to the saline-infused groups. Together, these results showed that maintaining a high level of inhibition in the striatum, in other words, preventing the transient decrease in inhibition that accompanies learning (see Figure 2), was sufficient to prevent task acquisition in control animals.

## Discussion

In the current study, we first verified the role of the corticostriatal pathway in auditory learning. Through chemogenetic suppression of excitatory input from AC layer 5 to the auditory striatum, we showed that learning was significantly delayed when corticostriatal suppression was maintained across all testing days (Figure 1C, purple line). Precisely, when suppression occurred early during learning, there was a significant impact on learning (Figure 1D, pink) as compared to late suppression. While those results do not exclude the involvement of additional downstream cortical and non-cortical areas in auditory associative learning (e.g., thalamus, prefrontal cortex, and hippocampal regions: Pasupathy and Miller, 2005; Hart et al., 2018; Le Merre et al., 2018), our results are in line with previous studies using optogenetic manipulation of striatal cells (Znamenskiy and Zador, 2013; Xiong et al., 2015; Guo et al., 2018; Chen et al., 2019). Here, we also found a small improvement in behavioral d′ values when the corticostriatal pathway was suppressed, although d′ never rose beyond 1.0. This may suggest that other circuits, like the direct thalamic drive to the striatum, may also in part contribute to enhancing performances (Ponvert and Jaramillo, 2019). In addition, despite limiting the chemogenetic manipulations to layer 5 AC cells and selectively targeting the projections to the auditory striatum, input from other cortical layers (e.g., layer 2/3; Yamashita et al., 2018) may also have been suppressed.

While AC layer 5 IT and PT cells showed significant increases in inhibitory strengths during the acquisition phase, striatal D1 and D2 MSNs presented significant decreases in inhibitory strengths (Figure 5). A reduction of inhibitory synaptic gain has often been linked to associative learning. For instance, fear conditioning was found to be associated with the inhibition of parvalbumin-positive layer 2/3 interneurons in the AC (Letzkus et al., 2011; Sarro et al., 2015) and interneurons in the amygdala (Wolff et al., 2014). Similarly, reduced inhibitory strengths have also been associated with motor learning (Smyth et al., 2010; Baarbé et al., 2014; Coxon et al., 2014). Our findings for striatal D1 and D2 cells support the idea that a reduction of inhibition is a general mechanism involved in many forms of associative learning. In addition, our results suggest that co-activation of both the direct and indirect pathways may contribute to enhancing auditory discrimination performance. Conversely, the increase in inhibition in AC layer 5 IT and PT cells may potentially gate sensory information during task acquisition, in order to potentiate relevant cues and attenuate irrelevant sensory noise (Egger et al., 2020). Although we have attempted to classify D1 and D2 cells in the current study, there is a large overlap in the different physiological properties of those cells (Goodliffe et al., 2018). Since our results showed similar changes in both D1 and D2 cells, it is safe to consider that our classification did not impact the results. Although we did not directly test the interdependence of the cortical and striatal changes, the latter may potentially support LTP through the strengthening of different subsets of corticostriatal connections in order to elicit the Go response and the Nogo response.

We next assessed how the corticostriatal pathway supports learning in animals which had a transient period of developmental auditory deprivation. In line with Mowery et al. (2017), striatal dysfunctions were shown to persist long after the actual period of sensory deprivation. Indeed, inhibitory strengths and firing rates in striatal D1 and D2 cells were significantly lower as compared to the control population. While a transient reduction of inhibitory strengths of striatal cells was found during learning in the control group, in contrast for the EP striatal cells, augmented inhibition accompanied learning, and the firing rates of EP striatal cells approached control values during task acquisition (Figure 3, and Supplementary Figure 4). In addition, IPSP amplitudes of AC IT and PT cells of EP animals were higher than for control animals at the naïve stage, and learning was not accompanied by a change in IPSP amplitudes. This suggests that the increased inhibition seen for the control animals during learning, potentially for noise reduction purposes at the cortical level, was already present in the EP animals. Together, these results suggest that instead of reduced inhibition, a certain range of synaptic inhibition values, implying a certain balance of excitation and inhibition (Froemke, 2015), may be crucial for plasticity to occur. Such transient shifts in inhibitory synaptic strengths during learning in the control and EP animals may be required in order to achieve such an optimal state for plasticity.

The inhibitory and firing rate changes observed for the EP animals during learning could be further explained by phenotype-specific forms of cellular physiology compensation (Supplementary Figure 5). Changes in resting membrane potential, membrane resistance, and sensory adaptation allowed the direct and indirect pathway neurons to briefly achieve control level firing rates during task acquisition. D1 cells in the EP animals presented a brief increase in sensory adaptation ratio at all stimulation levels, and D2 cells presented increased intrinsic excitability through transiently more depolarized resting membrane potentials and a brief increase in membrane resistance. Such transient changes in intrinsic properties may enhance the probability of eLTP along the corticostriatal circuit and in downstream areas, that manifest behaviorally as an improvement in discrimination performances. Thus, through such phenotype-specific compensatory mechanisms, the acquisition of a Go-Nogo discrimination task in the EP animals was similar to control animals.

However, in more complex tasks (e.g., several Go and Nogo stimuli, closer modulation rates Go and Nogo stimuli) or poorer signal to noise conditions (e.g., in a noisy environment), EP animals may present significant learning deficits (e.g., perceptual learning deficits, see Caras and Sanes, 2015). In humans, transient hearing loss is associated with behavioral deficits that can outlast the period of elevated hearing thresholds (Pillsbury et al., 1991; Hall and Grose, 1994; Hogan et al., 1996; Hall et al., 1998; Hogan and Moore, 2003). Children presenting repeated episodes of ear infection (otitis media-related hearing loss) have been shown to have auditory processing and language impairments, even though audibility is normal at the time of testing (Hall et al., 1995; Whitton and Polley, 2011). Thus, the transient developmental sensory deprivation used here represents a good model to study changes in circuit dynamics both when the behavioral performance is impacted and in conditions of control-like behavioral performance.

Overall, the current study provides a better understanding of how the corticostriatal pathway supports auditory learning through transient inhibitory shifts in striatal D1 and D2 MSNs, governed at least in part by GABAA-α2/3 containing receptors (Figure 5). Those findings are of broad importance as the etiology of many neurological disorders is linked to abnormal synaptic set points of GABAA receptor-mediated inhibition (diminished GABAA in epilepsy: Treiman, 2001; autism: Chao et al., 2010; tinnitus: Richardson et al., 2012; fragile X syndrome: Braat and Kooy, 2015; increased GABAA in Down syndrome: de San Martin et al., 2018; Schulz et al., 2019; Huntington’s Disease: Holley et al., 2019). In addition, chronic imbalance in cortical supragranular excitatory/inhibitory tone through diminished GABAA receptor-mediated inhibition is a common feature of developmental sensory deprivation (vision: Maffei et al., 2006; somatosensory: Jiao et al., 2006; auditory; Takesian et al., 2009; Mowery et al., 2014). Up or downregulation of GABAA-α1 containing receptors has previously been shown to govern mature forms of inhibitory synaptic transmission (Fritschy et al., 1994; Heinen et al., 2004; Bosman et al., 2005). Hence, restoration of GABAergic inhibition in cases of behavioral deficits could be a valuable target to investigate for potential therapy approaches (Verret et al., 2012; Schmid et al., 2016; Dargaei et al., 2018; Mowery et al., 2019).

## Data Availability Statement

The original contributions presented in the study are included in the article/Supplementary Material, further inquiries can be directed to the corresponding author.

## Ethics Statement

The animal study was reviewed and approved by University Animal Welfare Committee at New York University.

## Author Contributions

NP and TM designed the experiments, performed the experiments, analyzed the data, and wrote the article.

## Conflict of Interest

The authors declare that the research was conducted in the absence of any commercial or financial relationships that could be construed as a potential conflict of interest.

## Abbreviations

AM: amplitude modulation
AC: auditory cortex
DREADD: Designer Receptors Exclusively Activated by Designer Drug
EP: earplugged-reared
EPSP: excitatory postsynaptic strengths
IPSP: inhibitory postsynaptic strength
IT: intratelencephalic neurons
eLTP: long-term excitatory plasticity
MSN: medium spiny neuron
PT: pyramidal tract neurons.
