# Supplementary material for: Early Sensory Deprivation Leads to Differential Inhibitory Changes in the Striatum During Learning
Source: Front Neural Circuits. 2021 May 28;15:670858. doi: 10.3389/fncir.2021.670858 (PMC8194259; doi:10.3389/fncir.2021.670858)
Supplement: Supplementary file 1 [file Presentation_1.pdf]

Title: **Early sensory deprivation leads to differential inhibitory changes in the striatum during learning**

Authors: Nihaad Paraouty & Todd M. Mowery

SUPPLEMENTARY MATERIAL

SUPPLEMENTARY FIGURES 1-5 & FIGURE LEGENDS

SUPPLEMENTARY INFORMATION

The inhibitory effect of iDREADD was verified *in vitro* by using a functional corticostriatal slice preparation (Supplementary Figure 2). After transfection (~3 weeks), corticostriatal slices were obtained and whole cell recordings from D1 and D2 cells in zones of fluorescently labeled layer 5 terminals were carried out (Supplementary Figure 2B, far right plots). Biphasic electrical stimulation of AC layer 5 was used to establish baseline EPSPs, followed by 30 minute bath application of the activating drug, c21. Post drug measures were taken at 10 minutes interval up to an hour. There was a significant decrease in evoked EPSP amplitudes of MSNs when stimulating AC (Supplementary Figure 2C, yellow traces), however, no change was found when stimulating the thalamus (Supplementary Figure 2C, brown traces; repeated measures ANOVA: $F_{1,1}=5740.41$, $p<0.0001$). For both AC and thalamic stimulation, the EPSP measures after washout were not significantly different as compared to baseline values (post-hoc comparisons: AC: $p=0.3287$; thalamus: $p=0.9910$). These results suggest that the auditory inputs from the thalamus directly to the striatum were also preserved in vivo, and confirm that the learning differences in vivo were due to the suppression of solely the corticostriatal pathway.

SUPPLEMENTARY FIGURE 1

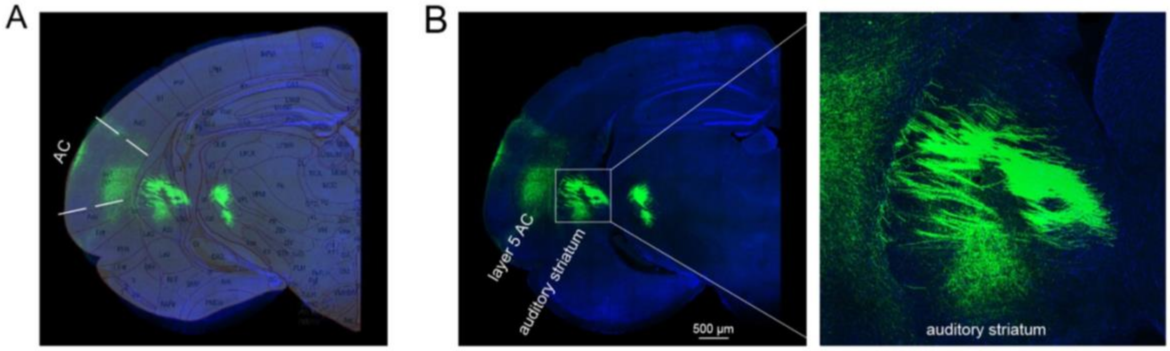

Corticostriatal projections from auditory cortex to auditory striatum. **A.** Coronal view of the primary auditory cortex (AC) and its cortical inputs to the auditory striatum. The corresponding figure from the gerbil atlas (Radtke-Schuller et al., 2016) is superposed. **B**. Brain slices showing the injection site in layer 5 AC and anterograde labeling in the striatum. Expanded inset of the striatum showing dense axonal labeling.

SUPPLEMENTARY FIGURE 2

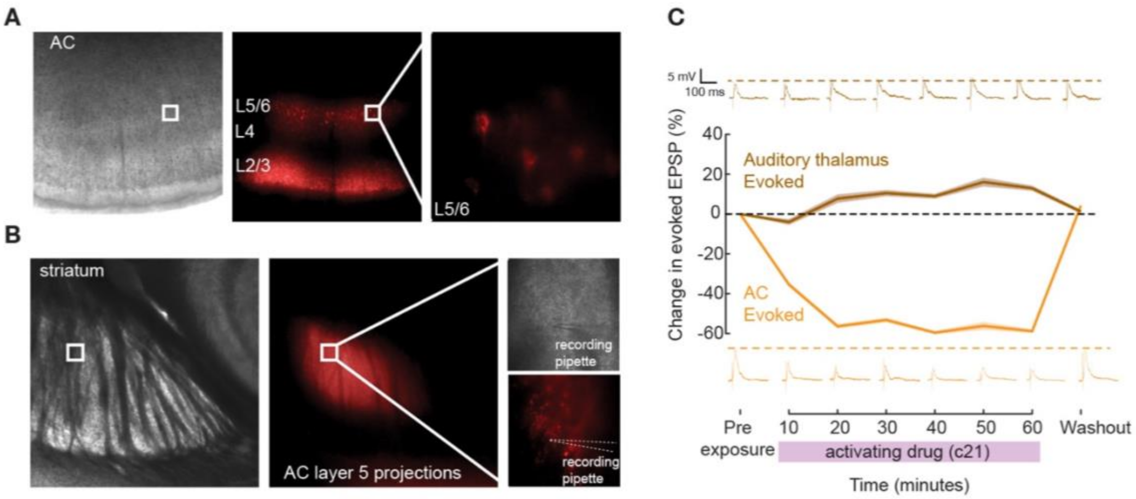

Targeting only corticostriatal projections and not thalamostriatal projections. **A**. Brightfield images (left) from AC, showing pyramidal neurons expressing mCherry. **B**. Brightfield images (left) from auditory striatum, showing medium spiny neurons expressing mCherry from AC layer 5 projections. The right panels show a patched medium spiny neuron receiving synaptic terminals from transfected AC layer 5 projections. **C**. Individual excitatory postsynaptic (EPSP) traces recorded from MSNs, before and after the activating drug (c21) was added to the bath. Top traces (in brown) show EPSP individual traces and mean change (n=6 cells; mean±sem) in evoked EPSP when electrically stimulating the thalamus. Bottom traces (in yellow) show EPSP individual traces and mean change (n=6 cells; mean±sem) in evoked EPSP when the electrically stimulating the AC.

Supplementary Figure 3

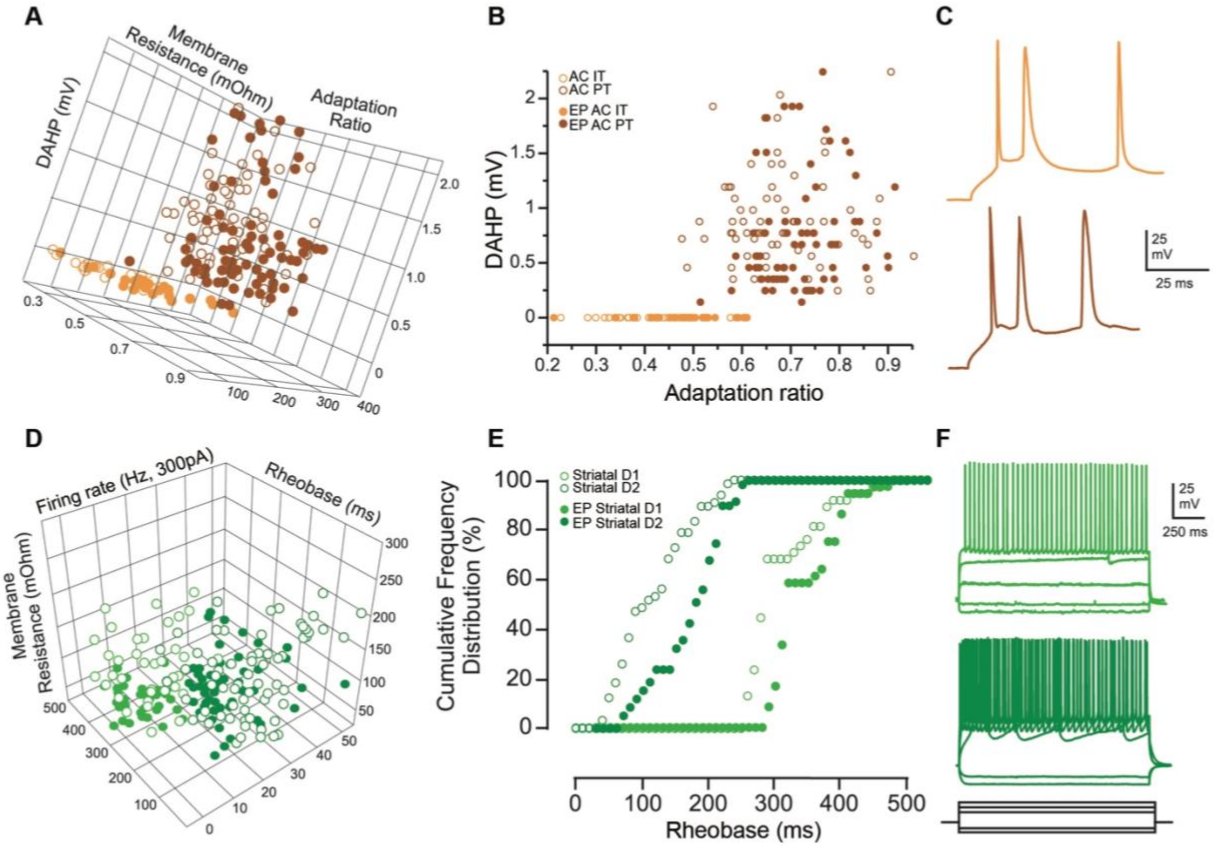

Classification of striatal D1 and D2 MSNs and AC layer 5 IT and PT cells. **A**. Cellular physiology of layer 5 AC cells plotted as a function of sensory adaptation ratio, membrane resistance (mOhm), and depolarizing voltage after hyperpolarization (DAHP, mV) for both the control and the EP animals.. **B**. Adaptation ratio and DAHP (mV) plot of AC layer 5 cells for the control and EP animals. **C**. Representative traces of an IT and a PT cell from the control group. **D**. Cellular physiology of striatal cells plotted as a function of membrane resistance (mOhm), firing rate (Hz, at 300pA) and rheobase (ms) for the control and the EP animals. **E**. Cumulative frequency distribution plot against rheobase (ms) for striatal cells in the control and the EP animals. **F**. Representative traces of a striatal D1 and a D2 cell from the control group.

SUPPLEMENTARY FIGURE 4

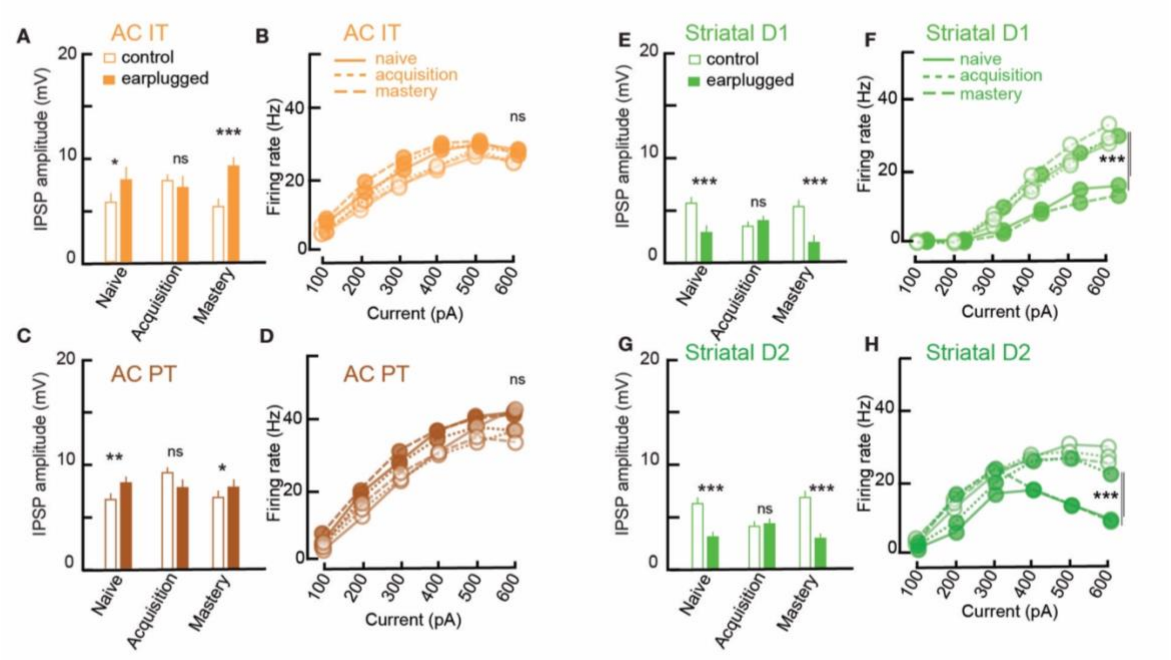

During task acquisition, striatal EP inhibition and firing rates move closer to control values. **A**. IPSP amplitudes of AC IT cells in the 2 populations (control and EP) during the three behavioral epochs. **B**. Firing rate patterns of AC IT cells. **C**. IPSP amplitudes of AC PT cells. **D**. Firing rate patterns of AC PT cells. **E**. IPSP amplitudes of striatal D1 cells in the 2 populations (control and EP) during the three behavioral epochs. **F**. Firing rate patterns of striatal D1 cells. **G**. IPSP amplitudes of striatal D2 cells. **H**. Firing rate patterns of striatal D2 cells. During the task acquisition phase, no significant difference was found between the control and EP group, in terms of IPSP amplitudes (of AC and striatal cells) and firing rates (of striatal cells).

SUPPLEMENTARY FIGURE 5

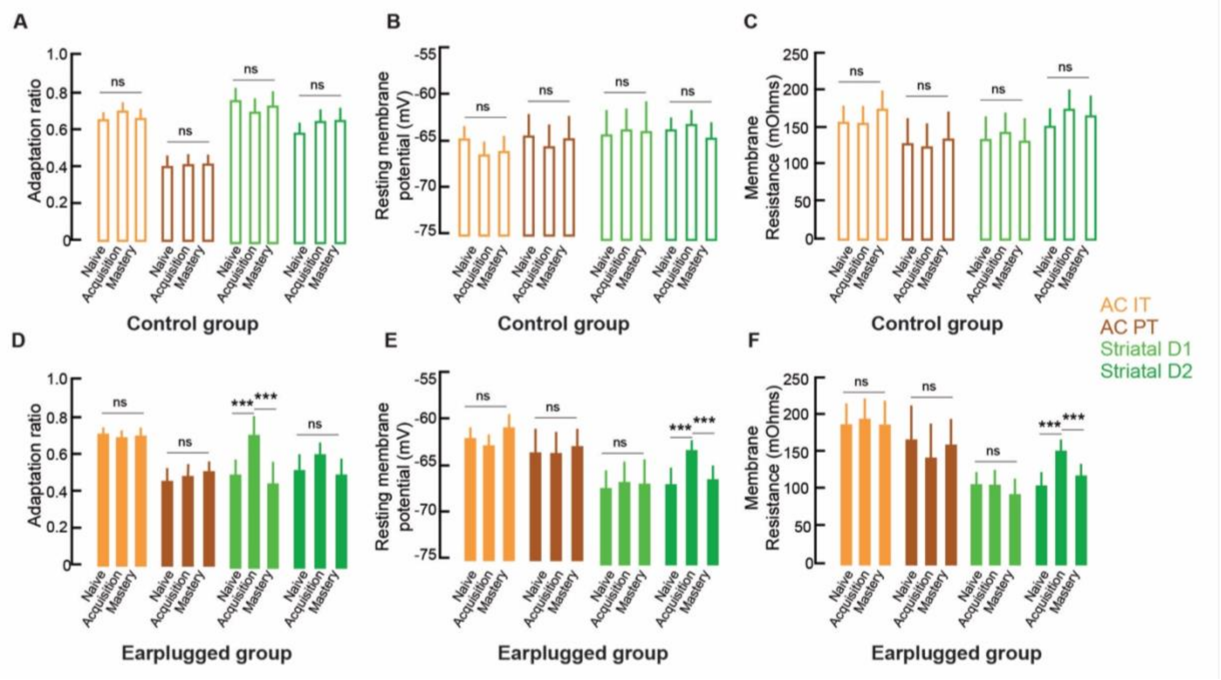

Phenotype-specific changes in cellular physiology of EP striatal cells. **A**. Adaptation ratio of all cells (AC IT and PT cells, and striatal D1 and D2 cells) during the three behavioral epochs in the control population. **B**. Resting membrane potential in the control population. **C**. Membrane resistance in the control population. No significant changes were found in the control population during learning. **D**. Adaptation ratio of all cells measured during the 3 stages of learning in the EP population. **E**. Resting membrane potential of all cells measured during the three behavioral epochs in the EP population. **F**. Membrane resistance in the EP population. EP striatal D1 cells showed a significant increase in adaptation ratio during task acquisition. In contrast, EP striatal D2 cells showed a significant increase in both resting membrane potential (more depolarized) and membrane resistance (higher) during task acquisition.
